# Characterizing the respiratory muscle strength of male British Army infantry recruits

**DOI:** 10.14814/phy2.70833

**Published:** 2026-06-05

**Authors:** N. C. Armstrong, S. J. Bailey, J. Osofa, W. Furby, A. Roberts, A. Rawcliffe, K. Hinde

**Affiliations:** ^1^ Human Performance Team, Defence Science and Technology Laboratory, Ministry of Defence Salisbury UK; ^2^ School of Psychology, Sport and Health Sciences University of Portsmouth Portsmouth UK; ^3^ Army Recruit Health and Performance Research, Medical Branch, HQ Army Recruiting and Initial Training Command, Ministry of Defence Upavon UK

**Keywords:** breathing, lung function, military, mouth pressure, soldier

## Abstract

This study aimed to quantify respiratory muscle strength in infantry recruits during the Phase 1 Combat Infantry Course (CIC) to identify if respiratory muscle weakness was present. Seventy‐six British Army infantry recruits volunteered to participate in the study with 36 participants completing the full data collection period. The strength of participants' inspiratory and expiratory muscles was assessed at weeks 1, 6, and 12 of the course. Mean inspiratory and expiratory muscle strength increased during the 12‐week course by 13.8% and 11.5%, respectively. However, of the recruits remaining in the course at week 12, ~20% produced inspiratory and expiratory values below their age predicted value. The exact cause of the increase in respiratory muscle strength is unknown. Likely contributory factors include an increase in volume and/or intensity of physical exercise and a training adaptation resulting from progressive load carriage training. It was concluded that there are individuals with respiratory muscle strength below age predicted values at the end of the 12‐week CIC; the impact of this finding on military task performance should be explored.

## Graphical abstract

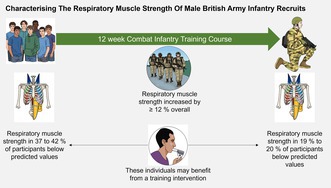

## 1 INTRODUCTION

Respiratory muscle strength is routinely measured in clinical and athletic populations to assess health and to optimize performance; it is used to distinguish disease from muscle weakness. Respiratory muscle weakness is detrimental to performance as it can limit lung inflation and exhalation, which reduces the volume of air available for gas exchange (American Thoracic Society/European Respiratory Society, 2002). Reference values have been developed to identify respiratory muscle weakness in both active and sedentary populations (Evans & Whitelaw, 2009). However, there are currently no respiratory muscle strength reference values for military populations. This knowledge gap is problematic because the ventilatory system of military personnel is exposed to stressors (e.g., load carriage, physical exertion, respirators, and altitude) that have a negative impact on performance (Armstrong et al., 2019; Faghy et al., 2016; Faghy & Brown, 2014; Phillips et al., 2016). Characterizing the respiratory muscle strength of military personnel will inform the implementation of training interventions designed to mitigate the effects of these stressors.

Comparisons between studies conducted in infantry soldiers (Armstrong et al., 2019) and those undertaken in trained civilians (Faghy et al., 2016; Faghy & Brown, 2014; Phillips et al., 2016) indicate that soldiers could have stronger respiratory muscles than their civilian counterparts, although it is accepted that a variation in the methods used between the studies may also be a contributing factor to differences found. The authors suggest that military tasks which place a strain on the ventilatory system may evoke a training adaptation to the respiratory muscles which enables personnel to perform in the battlespace despite this additional strain.

For example, torso borne load carriage increases the inertial (increased mass carried) and elastic forces (chest wall restriction) imposed on the torso which restrict shoulder elevation and chest wall expansion. This leads to a restrictive ventilatory impairment which is characterized by a reduction in forced vital capacity and forced expiratory volume in 1 s without a reduction in the ratio of these values (Armstrong et al., 2019). Furthermore, load carriage increases oxygen uptake requirements and energy expenditure when compared with unloaded exercise which creates additional ventilatory strain (Armstrong et al., 2019; Phillips et al., 2016, 2019). During load carriage, the wearer develops an inefficient breathing pattern (i.e., rapid and shallow) which reduces ventilatory reserve particularly during tasks with high ventilatory demands (Armstrong et al., 2019). Additionally, load carriage induces respiratory muscle fatigue (RMF) at moderate workloads (Armstrong et al., 2019; Faghy & Brown, 2014; Phillips et al., 2016). During unloaded exercise, RMF is typically observed at severe exercise intensities and leads to locomotor muscle fatigue and reduced performance of physical tasks (Harms et al., 1997; Romer et al., 2006).

The British Army Infantry Training Centre, Catterick (ITC(C)) delivers a 26‐week Combat Infantry Course (CIC) split into two phases (Phase 1: 12‐weeks; Phase 2: 14‐weeks). During Phase 1, load carriage is progressively introduced to recruits. The principle of training dictates that positive adaptations should occur with repeated load carriage exposure, and it has been shown that walking with backpack loads over a period of weeks decreases the energy cost of carrying load (Knapik et al., 2004; Wills et al., 2020), reduces the psychophysical demands of carrying a load (Wills et al., 2019) and increases aerobic fitness in military recruits (Harman et al., 2008; Wills et al., 2023). Soldiers who routinely carry torso‐borne loads likely experience some form of training adaptation and consequently experience less physiological strain compared with naive load bearers.

Therefore, the aim of this study was to quantify respiratory muscle strength in British Army Infantry throughout the CIC Phase 1 to identify (1) if respiratory muscle weakness was present at the start of the CIC and (2) if respiratory weakness was present at the end of CIC phase 1.

## 2 METHODOLOGY

This study was part of a larger study conducted by the Army Recruit Health and Performance Research Team, quantifying the physical demands (internal and external training loads, sleep characteristics), physical adaptations (body composition) and training outcomes (role fitness tests) of Standard Line infantry recruits over 12 weeks of Phase 1 CIC. The CIC aims to produce battle‐ready soldiers. During Phase 1 CIC, Standard Line infantry undergo a blended syllabus of generic military preparation and regimental specific soldiering skills, consisting of classroom‐based learning, foot‐drill, loaded marches, field exercises, weapon handling, and adventurous and physical fitness training.

### 2.1 Participants

Seventy‐six male recruits provided written informed consent to participate in the study in accordance with the Declaration of Helsinki (age: 21.4 ± 4.2 years; stature: 176.8 ± 7.3 cm; body mass: 74.5 ± 11.7 kg). No women enrolled in training at the time of this study. The study received a favorable opinion from the Ministry of Defence Research Ethics Committee (protocol:1041/MODREC/20). Participants had passed the initial Army medical evaluation and self‐reported respiratory tract infections. If participants experienced airway irritation or persistent coughing, they were excluded from the measurement and no data were recorded for that time point. Participants were permitted to participate in future test weeks if they were feeling well and no longer reporting symptoms of respiratory tract infections.

Data collection took place from August 2022 through to March 2023 in two cohorts. All physiological measurements were taken in an indoor environment. Temperature, humidity, and barometric pressure (mbar) were measured (Kestrel 5400 Heat Stress Tracker, US) alongside each physiological measurement. For Cohort One environmental measurements ranged from 16.1°C to 29.1°C, 39.7% to 74.6% humidity and 987.0 mbar to 1010.3 mbar. For Cohort Two environmental measurements ranged from 12.7°C to 20.1°C, 39.7% to 64.8% humidity and 971.0 mbar to 1010.5 mbar. Stretch stature was measured once at the beginning of basic training to an accuracy of 1 mm (Seca 213, GmbH & Co, Germany). Body mass was measured at each time point to an accuracy of 200 g using digital scales (Seca 875, GmbH &Co, Germany). Participants were asked to remove their footwear and were weighed in their standard clothing (t‐shirt and multi‐terrain pattern lightweight trousers).

### 2.2 Respiratory mouth pressures

The study used a repeated measures design. Measurements were taken during weeks 1, 6, and 12 of the CIC. Measurement of maximum inspiratory and expiratory mouth pressures (MIP and MEP, respectively) was achieved noninvasively using a calibrated handheld, respiratory mouth pressure meter (Micro RPM, Vyaire Medical, GmbH, Germany). This measure estimates the change in alveolar pressure; thus, mouth pressures represent the pressure generated by the respiratory muscles and the passive recoil of the respiratory system including the lung and the chest wall. If conducted in accordance with the appropriate control measures (American Thoracic Society/European Respiratory Society, 2002), the test–retest reliability of mouth pressure measurements is strong (Romer & McConnell, 2004). Preparatory work indicated the coefficient of variation for MIP and MEP was 2% to 6% and the intraclass correlation coefficient was ≥0.93 for PImax and ≥0.89 for PEmax when different investigators and different test days were considered (Lomax et al., 2026).

Measurements were made in accordance with the joint guidelines provided by The American Thoracic Society (ATS) and The European Respiratory Society (American Thoracic Society/European Respiratory Society, 2002) to ensure repeatability and reliability. In summary, participants wore a nose clip and adopted a standing position. They were instructed to fill/empty their lungs completely before inserting the mouthpiece and inhaling/exhaling maximally. The procedure lasted for a minimum of 1.5 s, with the highest mean pressure over 1 s recorded. Verbal encouragement was provided by the investigators. Three to eight measurements were taken until three measurements were obtained with a variation of less than 10%. The highest recorded measurement was used for analysis. A minimum of 1 min of recovery was given between attempts. To reduce the risk of a learning effect, participants were trained to a plateau (variation of <10%) in performance, as per the guidance provided by the American Thoracic Society (American Thoracic Society/European Respiratory Society, 2002).

### 2.3 Statistical analysis

The equations proposed by Evans and Whitelaw (Evans & Whitelaw, 2009), which account for sex and age, were used to determine predicted values and the lower limit of normal for respiratory muscle strength. The Evans and Whitelaw equations were selected as they combined data collected from multiple studies and included results from studies using only flanged mouthpieces due to ATS recommendations. In addition, the Evans and Whitelaw equations calculate the lower limit of normal rather than set an arbitrary value (e.g., two standard deviations from predicted normal value).

Data were analyzed in R version 4.1.1 (R Core Team, 2021) using a number of packages (tidyverse v2.0.0, dplyr v1.1.2, plyr v1.8.8, lubridate v1.9.2, ggplot2 v 3.4.2, lme4 v1.1‐31, lmrTest v3.1‐3, emmeans v1.10.6, and ggbeeswarm v0.7.2). Data were checked for outliers and removed if z‐scores were <−3 or >3. A linear mixed effects model was used to detect differences between weeks for respiratory muscle strength. The model structure included fixed effects for time, with participant as the random effect to account for individual variability. Alpha (α) was set to 0.05 for all comparisons. Assumptions of normality were checked using the model residuals and visual inspection of Q‐Q plots and histograms. Bonferroni adjusted pairwise comparisons were computed when a significant main effect was reported. Where values are provided, they represent mean ± standard error unless stated otherwise. Percentage change data was calculated: [(final value–initial value)/initial value] × 100.

## 3 RESULTS

Thirty‐six participants completed the study. Thirty‐nine participants withdrew from the CIC and one participant withdrew from the study. In addition, some participants were unable to complete all three test sessions because they did not meet the study inclusion criteria (e.g., persistent cough) at a given time point. As such, the withdrawals and exclusions of participants resulted in 73, 47, and 36 measurements recorded for weeks 1, 6, and 12, respectively. No difference in body mass was observed during the study (*F*_(2,88.43)_ = 1.15, *p* = 0.3226, mean difference between weeks 1–6 = 0.55 kg, mean difference between weeks 6–12 = 0.01 kg).

### 3.1 Maximal inspiratory pressure (MIP)

**FIGURE 1:**
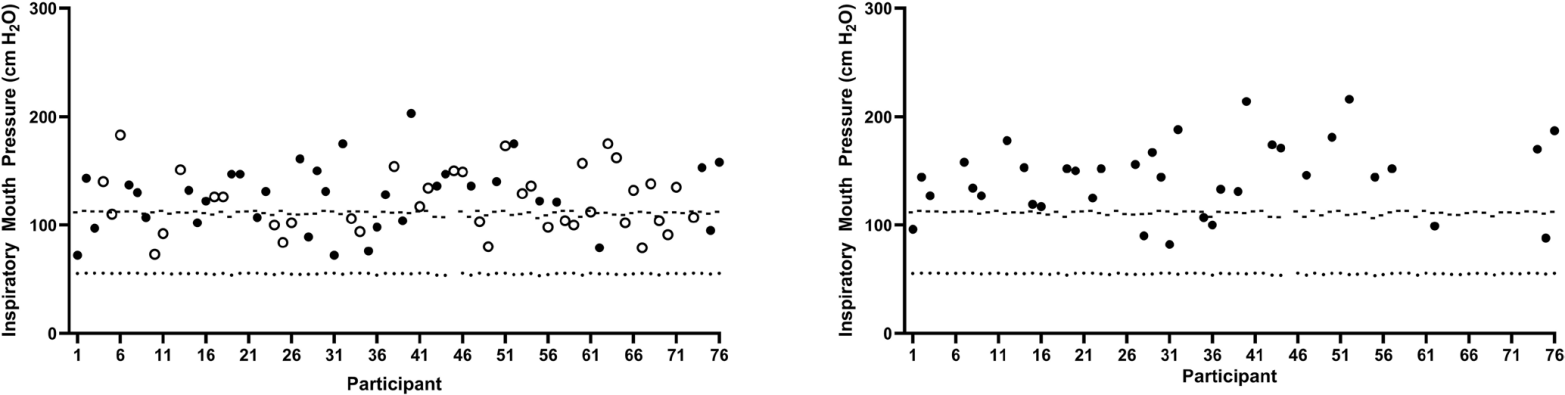
Individual MIPs at week 1 (left) and week 12 (right). Predicted values (−) and the lower limit of normal (.) are presented. Closed circles represent participants who completed CIC Phase 1; open circles represent participants who did not complete CIC Phase 1.

At week 1, 58% of participants achieved their predicted MIP values and 100% of participants produced values greater than their lower limit of normal (LLN). Of those remaining in training at week 12, 81% of participants achieved their age predicted MIP values, but all remained above their LLN (Figure 1).

**FIGURE 2:**
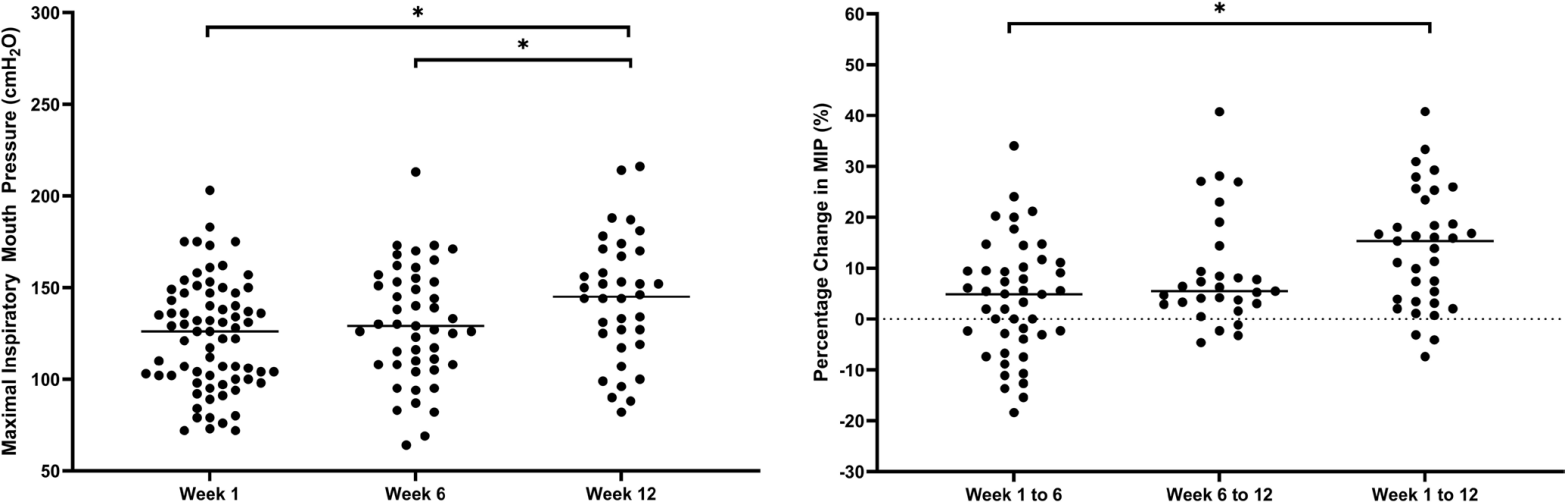
(Left) MIP at weeks 1, 6, and 12 of the CIC. (Right) Percentage change in MIP between weeks 1, 6, and 12 of the CIC.*Denotes a significant difference (*p* < 0.001). Solid thick black line represents mean. Line at 0 represents no change in MIP.

When considering group means, a main effect of time was observed for MIP (*F*_(2,82.1)_ = 29.48, *p* < 0.001). Post hoc testing revealed an increase in MIP in week 12 (140.6 ± 3.9 cm H_2_O) compared to week 1 (124.2 ± 3.6 cm H_2_O, *p* < 0.001, *d* = 0.57, 95% CI for difference = 11.12 cm H_2_O to 21.58 cm H_2_O) and week 6 (128.6 ± 3.8 cm H_2_O, *p* < 0.001, *d* = 0.48, 95% CI for difference = 6.46 cm H_2_O to 17.43 cm H_2_O) (Figure 2). A significant main effect of time was reported for %ΔMIP (*F*_(2,79.15)_ = 8.33, *p* < 0.001). Figure 2 shows the significant difference identified was the %change between weeks 1–12 (13.8%, 95%CI: 10.0%–17.6%) being greater than the % change between weeks 1–6 (3.8%, 95%CI: 0.5%–7.1%) (*p* = 0.0003).

### 3.2 Maximal expiratory pressure (MEP)

**FIGURE 3:**
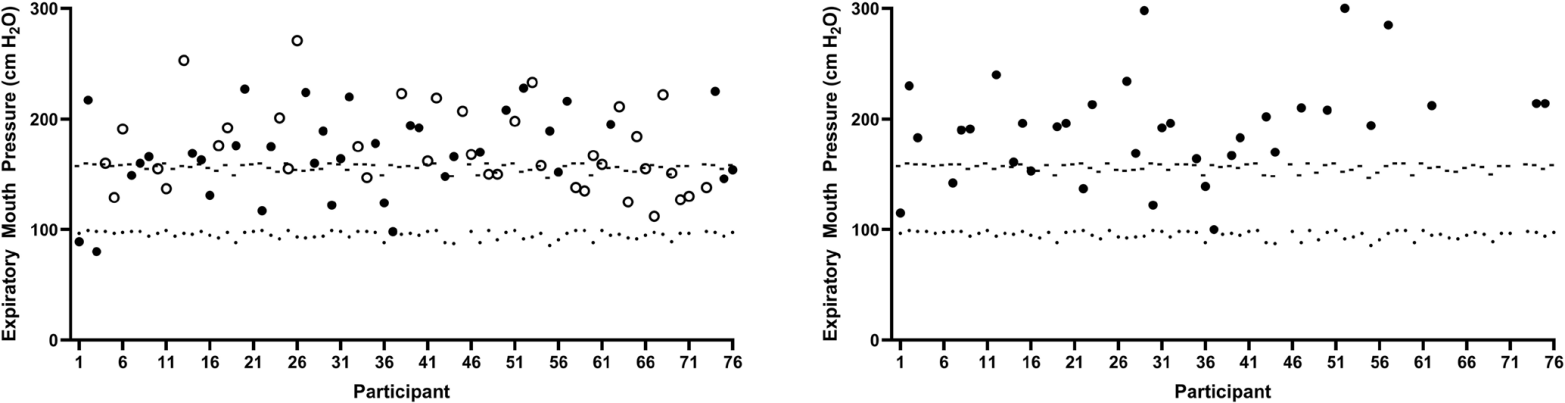
Individual MEPs at week 1 (left) and week 12 (right). Predicted values (−) and the lower limit of normal (.) are presented. Closed circles represent participants who completed CIC Phase 1; open circles represent participants who did not complete CIC Phase 1.

At week 1, 63% of participants achieved their predicted MEP values and 96% of participants produced values greater than their LLN. Of those remaining in training at week 12, 80% of participants achieved their age predicted MEP values, and 3% of participants (*n* = 1) did not achieve their LLN (Figure 3).

**FIGURE 4:**
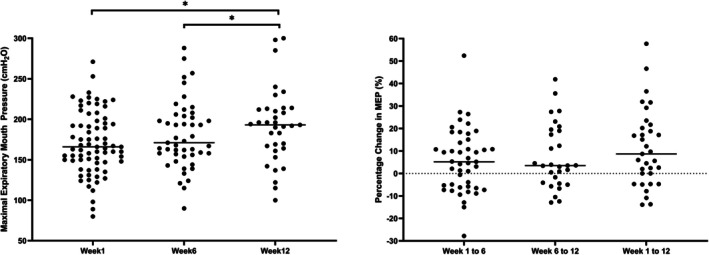
(Left) MEP at weeks 1, 6, and 12 of the CIC. (Right) Percentage change in MEP between weeks 1, 6, and 12 of the CIC.*Denotes a significant difference (*p* < 0.001) Solid thick black line represents mean, Line at 0 represents no change in MIP.

Analysis of group means identified a main effect of time for MEP (*F*_(2,85.97)_ = 10.20, *p* < 0.001) with post hoc testing revealing an increase in MEP in week 12 (191.2 ± 5.7 cm H_2_O) compared to week 1 (170.7 ± 4.7 cm H_2_O, *p* < 0.001, *d* = 0.53, 95% CI for difference = 9.30 cm H_2_O to 31.60 cm H_2_O, Figure 4). The %ΔMEP was similar across both halves of the course (*F*_(2,73.32)_ = 1.857, *p* = 0.163, Figure 4) with a total %change across the 12 weeks of 11.5% (95% CI = 6.4%–16.7%).

## 4 DISCUSSION

This study is the first to measure inspiratory and expiratory muscle strength in British Army recruits to investigate whether respiratory muscle weakness is present in recruits undertaking Phase 1 infantry training. Comparison of individual data with normal values developed for civilian populations revealed that 58% and 63% of individuals achieved their predicted values for MIP and MEP respectively at week one. Although this figure rose to 81% (MIP) and 80% (MEP) at week 12, there were still ~20% of individuals below their age predicted values at the end of the CIC Phase 1. The number of individuals that did not reach their lower limit of normal (LLN) for MEP was very low (*n* = 1) and all participants produced MIP values above their LLN.

The CIC is considered a physically demanding course within the British Army, with the main aim of transforming civilians into battle‐ready, trained soldiers. From 2021, new recruits followed an updated programme which involved a reduction in group running mileage and a focus on self‐paced exercise (e.g., running and load carriage), high intensity interval training, and strength and conditioning. In addition, a more progressive nature with regards to the physical aspects of the course have been employed, especially during the initial weeks.

Exploratory analysis on grouped data was conducted to inform future work. The analysis revealed that both inspiratory and expiratory muscle strength increased during the CIC. MIP increased by 13.8% and MEP increased by 11.5%, with 91% and 76% of individuals demonstrating improvements respectively. The exact cause of this increase in respiratory muscle strength is unknown. However, a contributory factor could be the increase in the amount and intensity of unloaded physical exercise performed by the recruits compared to upon entry resulting in improved aerobic performance and stronger respiratory muscles (Dunham & Harms, 2012; Fabrin et al., 2023; Segizbaeva & Aleksandrova, 2021). Unpublished results from the wider study indicate a larger percentage of time spent in higher heart rate zones during the latter weeks of the course and an improvement in unloaded 2 km run time (mean reduction of 52 s) in week 12 compared with week 1.

Another factor contributing to the increase in MIP and MEP could have been a training adaptation developed as a result of load carriage training (Armstrong et al., 2019). The physical development programme for the CIC showed that within the first 5 weeks (i.e., the first half of basic training), physical fitness training focused mainly on strength and conditioning, with one load carriage session in week 4. Whilst training in the latter weeks involved a greater number of load carriage sessions, with heavier weight carried. The percentage improvement in MIP from weeks 1 to 12 supports the theory that training with a load may act as a similar type of ergogenic aid to inspiratory muscle training (Armstrong et al., 2019), although increases in inspiratory muscle strength seen in this study were unsurprisingly smaller than those reported following dedicated inspiratory muscle training programmes (25%–30%) (Faghy & Brown, 2016; Griffiths & McConnell, 2007; Romer et al., 2002).

Greater increases were seen with MIP than MEP, with MIP more consistently improving (only 3 individuals showed no improvement at week 12). Significant increases in MIP in the absence of changes in MEP have been reported following endurance and high intensity interval training programmes (Dunham & Harms, 2012). Similarly, less pronounced changes in MEP are reported following inspiratory and/or expiratory muscle training programmes (Griffiths & McConnell, 2007). The ventilatory strain imposed by load carriage is greatest during inspiration, which accounts for greater increases in MIP in those who regularly carry torso borne load.

It is unclear what the repercussive military performance consequences are when respiratory muscle strength is below predicted normal values. Whilst the current study suggests that respiratory muscle strength does not limit CIC Phase 1 pass rates, there is strong evidence in civilian populations that strengthening the respiratory muscles is beneficial for loaded exercise performance (Faghy & Brown, 2016) and could have greater benefit for military populations in Phase 2 when greater loads are carried during training. As such, individuals close to their LLN may observe performance benefits with a training intervention. The benefits of respiratory muscle training in UK military populations have not yet been investigated. However, it is hypothesized that inspiratory muscle training could alleviate some of the detrimental consequences that loaded marching has on the ventilatory system (e.g., RMF) and subsequent negative perceptual effects. Furthermore, the role of the respiratory muscles extends beyond breathing and includes the maintenance of posture and stability (Brown & McConnell, 2012). As such, the benefits of respiratory muscle training may extend beyond task performance to the health and safety of military load carriers.

Torso‐borne load carriage involving moderate intensity exercise impairs ventilatory responses (Armstrong et al., 2019; Faghy et al., 2016; Faghy & Brown, 2014; Phillips et al., 2016), which changes an individual's breathing pattern, becoming faster and shallower (Armstrong et al., 2019), leading to psychophysical measures being negatively impacted (e.g., increased perceived exertion, perceived breathing effort and discomfort) (Armstrong et al., 2019). Changes in ventilation can lead to respiratory muscle fatigue (RMF) which accelerates the onset of peripheral fatigue in locomotor muscles, additional recruitment of accessory respiratory muscles, greater perceived breathlessness and exertion, and ultimately leads to early termination of exercise (Romer & Polkey, 2008). Increases in respiratory muscle strength following inspiratory muscle training have been shown to improve time trial performance in cycling (Romer et al., 2002), running (unloaded (Edwards et al., 2008) and loaded (Faghy & Brown, 2016, 2019)) and rowing (Griffiths & McConnell, 2007). Stronger respiratory muscles have also been reported to be positively correlated with shooting performance in police cadets (Karaduman et al., 2022). Only one study to date has employed military personnel to investigate whether inspiratory muscle training improves physical performance. Sperlich et al. (Sperlich et al., 2009) conducted a 6‐week intervention using German Special Forces Police, but results did not show any improvements in MIP, MEP, V̇O_2max_ or endurance performance (e.g., running speed at lactate threshold). There were no performance tests involving load carriage despite the authors stating that members of special force units undertake demanding physical exercise with heavy loads. As such, the effect of such training on load carriage performance was not accounted for. Stronger respiratory muscles may lessen or delay RMF experienced during load carriage and result in optimized performance, although this has yet to be evidenced in military populations and/or investigated using meaningful military relevant tasks.

### 4.1 Limitations

No women began training at the start of this study. When matched for height to their male counterparts, women have smaller airways, a decreased capacity for lung diffusion, and weaker respiratory muscles (Leahy et al., 2023). However, there is evidence that women may be less susceptible to diaphragmatic fatigue (Leahy et al., 2023). Given these sex differences, it is important that future work strives to document changes in respiratory muscle strength in both sexes. Furthermore, potential interventions must be assessed in both sexes.

Mouth pressure measurements are volitional tests that rely on sufficient training and motivation of the participant. There are non‐volitional methods for assessing respiratory muscle strength; however, they are more invasive and require clinical settings. A global index of respiratory muscle strength, which includes more muscles than just the diaphragm, was deemed appropriate for addressing the study aims. In addition, verbal encouragement for each measurement was provided to increase participant motivation.

The authors acknowledge that participants may have become familiarized with the measurements over the three time points, which may account for an increase in respiratory muscle strength over time. However, additional time was allocated at the start of the study to ensure participants were trained and achieved a plateau in performance, to reduce the risk of a learning effect.

The limitations associated with conducting data collection in a field environment apply to this study. As this study was conducted alongside the CIC, the authors were unable to control for factors such as time of day, sleep deprivation, and environmental conditions. These factors were recorded by the investigators but were not controlled for and may account for reductions in RMS that were observed in some individuals during the 12 weeks. However, it should be acknowledged that military personnel are routinely exposed to operational stressors. These stressors may cause a reduction in performance below their normal performance baseline and personnel must still achieve mission success despite these stressors.

## 5 CONCLUSION

This work has identified that the respiratory muscle strength of some recruits undertaking the CIC phase 1 is below aged predicted normal values. Increases in inspiratory and expiratory muscle strength occur in most (but not all) recruits during CIC phase 1. Future work should explore if there are military task performance consequences associated with respiratory muscle weakness. Further, the potential health and performance benefits of a training programme designed to increase the strength of the respiratory muscles should be explored in military personnel.

## AUTHOR CONTRIBUTIONS

**N. C. Armstrong:** Conceptualization; data curation; formal analysis; funding acquisition; investigation; methodology; project administration; resources; supervision; visualization. **S. J. Bailey:** Investigation. **J. Osofa:** Investigation. **W. Furby:** Investigation. **A. Roberts:** Funding acquisition; methodology; project administration; resources. **A. Rawcliffe:** Investigation; project administration. **K. Hinde:** Data curation; formal analysis; investigation; project administration.

## FUNDING INFORMATION

This work was funded by the Ministry of Defence through the Dstl Optimising Human Performance Project.

## CONFLICT OF INTEREST STATEMENT

There are no competing interests.

## ETHICS STATEMENT

This study received a favorable opinion from the Ministry of Defence Research Ethics Committee (protocol:1041/MODREC/20).
